# Policy barriers to drug repurposing in Europe: different stakeholder perspectives identified during survey-based shortlisting of key challenges

**DOI:** 10.1016/j.hpopen.2026.100168

**Published:** 2026-03-25

**Authors:** Kristóf Gyöngyösi, Zsuzsanna Ida Petykó, Dalma Hosszú, Pan Pantziarka, Helene G. van der Meer, Donald C. Lo, Marcell Csanádi, George Dennis Obeng, Zoltán Kaló, András Inotai

**Affiliations:** a Center for Pharmacology and Drug Research & Development, Semmelweis University, Budapest, Hungary; b Center for Health Technology Assessment, Semmelweis University, Budapest, Hungary; c Syreon Research Institute, Budapest, Hungary; d Anticancer Fund, Meise, Belgium; e ZonMw, The Hague, the Netherlands; f European Infrastructure for Translational Medicine (EATRIS), Amsterdam, the Netherlands; g Syreon Research Africa, Accra, Ghana

**Keywords:** Drug repurposing, Off-patent, Pricing and reimbursement, Multi-stakeholder, Barriers, Market authorisation

## Abstract

**Background:**

Finding new therapeutic indications for established medicines continues to have important impact. Although drug repurposing (DR) offers the potential for a faster and more affordable complement to *de novo* development, barriers to successful DR remain pervasive.

**Objective:**

To prioritize barriers identified earlier in a systematic literature review by the REMEDi4ALL Horizon Europe project, involving multiple stakeholder groups, to create a shortlist of the most important barriers and to examine differences in stakeholders’ perceptions.

**Methods:**

A policy survey was conducted among stakeholder groups involved in DR. Participants rated the barriers based on their impact and actionability, using 5-point categorical scales. A weighted scoring method was used to create the shortlist by combining scores across domains, while ensuring that each stakeholder group’s preferences were retained in the final shortlist.

**Results:**

60 individual responses were collected. The final shortlist contained 22 barriers, including 4 barriers related to exclusivity rights for repurposed medicines (RMs), 2 to pricing of RMs, 5 to market authorization of RMs, 2 to perception off-patent RMs, 2 to business case for off-patent RMs, 2 to non-industry funded DR, 2 to health technology assessment of RMs, 2 to ecosystem for non-profit or small-medium sized enterprises-driven DR, and 1 to business case for repurposing on-patent compounds.

**Conclusion:**

Prioritizing barriers helps identify solutions by addressing the critical challenges first. Acknowledging that different stakeholder groups may perceive the impact and actionability of these barriers differently is crucial for building a shared, multi-stakeholder perspective when formulating policy recommendations to address the barriers.

## Highlights

• Drug repurposing faces multiple policy barriers.
• Prioritizing policy-related barriers hindering drug repurposing.
• A shortlist of the most important barriers contains 22 barriers.
• Prioritizing barriers enables targeted development of recommendations.
• Recognizing differing stakeholder views is vital for building consensus.

## 1 Introduction

Drug repurposing (DR) – the process of identifying new therapeutic uses for existing medicines (approved or investigational stage) beyond their original indication – has emerged as a promising complement to *de novo* pharmaceutical development, by offering an affordable and time-efficient approach [1], [2], [3]. It has gained significant attention recently, further amplified by network medicine [4], [5]. Publicly available databases can also support non-commercial DR activities [6], [7], [8]. It is especially useful for rare and ultra-rare diseases, where no other effective treatment options exist [9]. Nevertheless, the full potential of DR has not yet been utilized, mainly due to current hurdles in the regulatory system and the lack of a business case for repurposing off-patent medicines [10]. Due to the emerging significance and need of DR, the European Union funded numerous projects across the Horizon Europe program over the recent years. These include the Precision drug REPurpOsing For EUrope and the world (REPO4EU) [11] and REpurposing of MEDicines 4 ALL (REMEDi4ALL) [12] that are delivering expert services to institutionalize platforms to cater for the DR ecosystem – among many other initiatives [13], [14], [15], [16].

The research presented in this paper is conducted by authors working within the REMEDi4ALL consortium and builds on an earlier systematic literature review (SLR) in which the authors identified, structured, and validated policy barriers preventing the development of- and limiting patient access to on-label repurposed medicines (RMs) [17]. This SLR extracted 875 barrier mentions from 192 scientific documents. After deduplication of overlapping barriers, then applying thematic analysis, 32 policy barriers were identified. Subsequently, these were validated in 5 workshops (each lasting two hours) by 80 experts altogether, representing every involved stakeholder group in the DR ecosystem (e.g., health care payers, regulatory and health technology assessment (HTA) experts, patient representatives, funders, researchers and representatives of the off-patent and originator pharmaceutical industry). After validation, the updated list contained 33 barriers that were categorized into 9 main themes and 20 subthemes.

These barriers serve as a starting point to explore potential areas for future policy recommendations by REMEDi4ALL. Discussions during the validation meetings also revealed that distinct stakeholder groups perceive these barriers differently. As a next step, prioritizing the 33 barriers supports efficient generation of policy recommendations by focusing on the most critical challenges first.

This study aimed to shortlist the barriers based on their perceived importance and to examine the stakeholder-specific differences on importance in greater detail.

## 2 Materials and methods

**Fig. 1:**
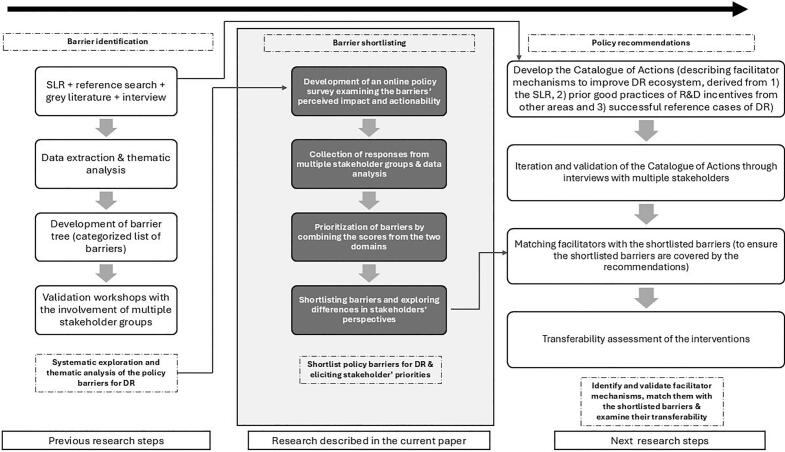
Complete Flowchart of the research by the REMEDi4ALL Consortium's Work Package 8. DR – drug repurposing; SLR – systematic literature review.

The overall policy research on barriers hindering DR, including the prior and the future steps to provide broader context, is summarized in Fig. 1.

### 2.1 Policy survey

#### 2.1.1 Structure

To elicit the perception of stakeholder groups regarding the importance of the identified barriers, we developed an online policy survey (Suppl. Table 1). The policy survey was implemented in Microsoft Forms. Survey responses were collected between the 6th of June and the 29th of July 2024. Participants were recruited from experts who attended the validation workshops and completed an online registration form circulated during REMEDi4ALL events (e.g. REMEDi4ALL Funders Network meetings and at the International Drug Repurposing Conference (iDR24)).

The first part of the policy survey asked for the participants' general information. To avoid the double counting of responses, responders' names, email addresses, and institutions were collected; however, analysis of the policy survey results was conducted anonymously. To capture regional nuances in perception and expertise, also in line with future transferability workstream, participants were asked to include the country they have relevant expertise for (i.e. where they work rather than where they are from). Finally, participants were requested to select their stakeholder group best describing their perspective from the following: Patient representatives; Researchers & academia; Funders of DR; Pharmaceutical, biotech and small-medium sized enterprises (SMEs), industry/association; Regulatory experts; Health technology assessment (HTA) experts; Health care payers; Clinicians & clinical experts, with an ‘Other − to be specified’ option provided.

In the main section, responders provided information regarding their perception of the listed barriers. Barriers were grouped by their main theme and examples from literature were given, to enhance understanding by responders. Rather than including an option to skip rating individual barriers, experts had the opportunity to skip an entire theme. The barriers were evaluated according to two separate domains: impact defined as *“Does the barrier have a strong negative effect on the development of repurposed medicines or these medicines reaching the target patient groups”* and actionability defined as *“How easy it is to overcome this barrier in the next 5 years at EU level? (Considering resources, current legislative, or policy frameworks and current practices).”* 5-point categoric scales were assigned to both domains for responders to indicate their perception on (1) impact: negligible, minor, moderate, major, extreme; (2) actionability: very poor, poor, average, easy, very easy. Finally, to ensure no barriers were missed, a comment section was inserted at the end of each theme, where participants could propose adjustments in the phrasing of the barriers.

#### 2.1.2 Processing and analysis of survey results

The policy survey results were processed and analysed using Microsoft Excel. Respondents who did not rate any of the barriers were excluded from the analysis. To ease shortlisting using responses to both domains, categorical scales were converted to a 0–4 scale, where the worst category was assigned 0 and the best category was assigned 4. The mean scores for both impact and actionability domains were calculated for each barrier. Impact and actionability scores were aggregated both by stakeholder groups (to have a better understanding of the perception of each stakeholder) and for the total cohort.

### 2.2 Shortlisting rules

#### 2.2.1 Main rules (aiming to narrow down the number of barriers)

To narrow down the number of barriers for a shortlist, the top 20 of 33 barriers were considered as a starting point. The cut-off was set as 20, since it was considered comprehensive while also showing the priority in terms of importance. Additionally, while both impact and actionability scores were considered for ranking, we prioritized impact to ensure that the most fundamental, system-level barriers to DR were included, even if difficult to address. Therefore, during the calculation of the overall score, impact was weighted twice as much as actionability:

Overall score = 2/3 * Impact score + 1/3 * Actionability score

#### 2.2.2 Additional rules (to ensure no stakeholder opinion is missed)

Three additional elements in the shortlisting methodology ensured that the unique preferences of all stakeholders were adequately represented and included in the final shortlist of barriers: (1) A priority list was created for each stakeholder group. If not represented within the overall (i.e. all stakeholder group’s) top 20 barriers, the top three barriers of each stakeholder group were added to the shortlist. (2) However, to prevent individuals from dominating the opinion of a stakeholder group (especially where the number of respondents within a group was small), barriers evaluated by less than 50% of respondents from that stakeholder group were not eligible to be added to the final shortlist. (3) Finally, aggregating impact by stakeholder group allowed us to capture differences regarding how each stakeholder group prioritized specific policy barriers to DR, reflecting their own perspectives. On the other hand, actionability was aggregated across the full cohort, as it would not have been meaningful to examine differences in how difficult each group perceived addressing the barriers to be. Therefore, the overall mean was used for the actionability domain instead of each stakeholder group’s mean, to calculate the composite scores for shortlisting:

Stakeholder score = 2/3 * Stakeholder Impact score + 1/3 * Overall Actionability score

The shortlisting methodology was presented to the REMEDi4ALL consortium and was approved at the REMEDi4All 2nd General Assembly Meeting in Budapest 17-18th October 2024. During this meeting the work on developing policy recommendations for the shortlisted barriers was initiated.

## 3 Results

### 3.1 Characteristics of policy survey participants and response rate

In total, we collected 63 individual responses; of which 60 were included in the analysis (3 were excluded because the participants did not provide any ratings for the barriers). Based on their initial selection in the survey, responders were clustered into 5 stakeholder groups (created by merging the stakeholder categories from the survey). In the survey the perspectives of both Old and New EU Member States were captured. Most of the experts indicated DR expertise/perspective relevant for Old EU Member States (EU15 countries excl. UK) (51.7%), the remainder were for New EU Member States (EU13 countries) (20%), the UK (16.7%), and Others (e.g., the USA, Switzerland and Ukraine) (11.7%). Characteristics of the participants can be seen in Suppl. Table 2.

The response rates for each theme are displayed in Supplementary Fig. 1. There was only one theme – *Exclusivity rights* with a response rate below 50% – completed by 29 experts. Perception of repurposing of off-patent medicines (88%) and Business case of repurposing off-patent medicines (75%) had the highest response rate. Importantly, no additional barriers were suggested in the comment section of the policy survey by responders.

### 3.2 Impact and actionability scores

**Fig. 2:**
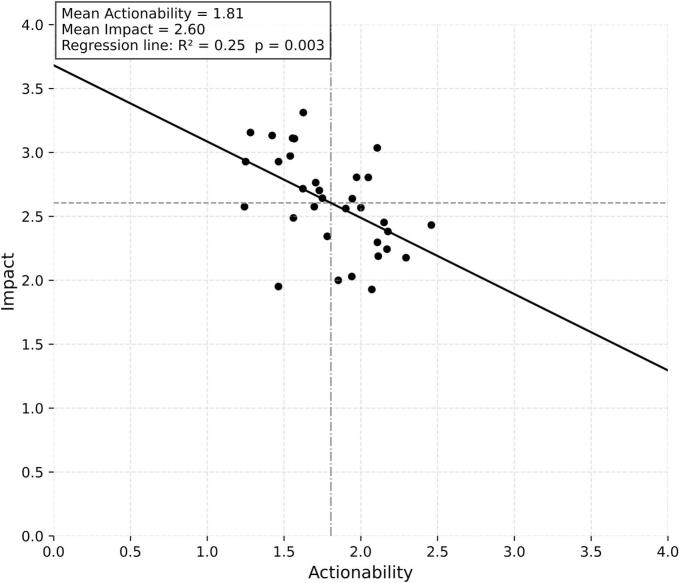
Impact and actionability scores of barriers (each dot represents a barrier).

The mean scores of each barrier can be seen in Fig. 2. The average score for actionability was 1.81 (out of maximum 4), while the average score for impact was 2.60. The score for actionability ranged from 1.24 to 2.45, while the score for impact ranged from 1.93 to 3.31.

Interestingly, a general negative relationship was observed between actionability and impact across all barriers (β = −0.60, R^2^ = 0.25, p = 0.003), suggesting that those perceived as having greater impact were often viewed as less actionable.

### 3.3 Overall shortlist

22 Barriers were included in the final shortlist: Beyond the initial list of the top 20, two additional barriers were ranked as high priority by the stakeholder group of patient representatives. The final shortlist of barriers can be seen in Table 1. The range of overall score between the first (2.75 out of 4) and last barrier (2.28 out of 4) on the shortlist was 0.47. Among the shortlisted barriers, 4 were related to exclusivity rights, 2 to pricing, 5 to market authorisation, 2 to perception of repurposing of off-patent medicines, 2 to business case for repurposing off-patent medicines, 2 to non-industry funded DR, 2 to HTA of RMs, 2 to ecosystem for non-profit or SME driven DR, and 1 to business case for repurposing on-patent compounds

**Table 1:** Shortlist of barriers.

Barrier	Theme	Total score (max. 4.0)	Impact (max. 4.0)	Actionability (max. 4.0)
Generic pricing mechanisms are often applied to off-patent repurposed medicines.	Pricing	2.75	3.31	1.63
Limited market protection and data exclusivity options for repurposed medicines.	Exclusivity rights	2.73	3.04	2.11
Limited incentives to turn off-label use to on-label to ensure access to a wider patient population.	Market Authorisation	2.59	3.11	1.57
Insufficient return on investment is anticipated for repurposing off-patent medicines.	Business case for repurposing off-patent medicines	2.59	3.11	1.56
Competitors can benefit from the DR investment in case of off-patent medicines by cross-label prescribing and dispensing.	Business case for repurposing off-patent medicines	2.56	3.13	1.42
Limited, incomplete and fragmented funding is available for non-profit DR at different stages.	Non-industry funded DR	2.55	2.80	2.05
Indication-based differential pricing for repurposed medicines is problematic.	Pricing	2.53	3.16	1.28
Evidence requirement for HTA is not designed for off-patent DR and is of high burden.	HTA	2.53	2.81	1.97
For label-extension, there is a need for marketing authorisation holder’s involvement for non-MAH developers.	Market Authorisation	2.50	2.97	1.54
There is a lack of clarity/limited awareness on evidence requirements for some off-patent drug repurposing cases.	Market Authorisation	2.44	2.93	1.46
Enforcement of market protection for repurposed medicines is difficult because of cross-label prescribing and dispensing.	Exclusivity rights	2.44	2.43	2.46
Lack of findable, accessible, interoperable, and reusable (FAIR) data (especially proprietary data) for DR.	Ecosystem for non-profit or SME driven drug repurposing	2.41	2.76	1.71
No tailored or predictable technology appraisal process exists for off-patent DR.	HTA	2.41	2.64	1.94
Evidence generation is burdensome for the market authorisation of off-patent medicines.	Market Authorisation	2.38	2.57	2.00
Incentives for market authorization of repurposed medicines in paediatric indications are not proportionate to the required efforts for evidence generation.	Market Authorisation	2.38	2.70	1.73
Enforcement of patent protection for repurposed medicines is difficult and costly.	Exclusivity rights	2.37	2.93	1.25
DR research of off-patent medicines is perceived as less-innovative, less robust or less attractive compared to de novo drug development.	Perception of repurposing of off-patent medicines	2.35	2.72	1.62
Cost of DR development is perceived to be disproportionally high compared to the risks and potential revenues.	Perception of repurposing of off-patent medicines	2.35	2.45	2.15
Limited options for patent protection of repurposed medicines.	Exclusivity rights	2.35	2.64	1.75
Public-private partnerships in funding DR are complex and not always possible.	Non-industry funded DR	2.34	2.56	1.90
The know-how needed for DR may not be available for non-profit entities or SMEs.	Ecosystem for non-profit or SME driven drug repurposing	2.31	2.38	2.18
Originator companies often lack incentives to repurpose on-patent compounds due to low expected return on investment and strategic business decisions regarding their disease portfolio.	Business case for repurposing on-patent compounds	2.28	2.58	1.70

DR – drug repurposing; HTA – health technology assessment; MAH – market authorisation holder; SME – small-medium sized enterprises.

### 3.4 Stakeholder priorities

The results of the stakeholder rankings (stakeholder heatmap) are presented in Table 2. Based on the preference of individual stakeholder rankings, two barriers were added to the final shortlist, coming from patient representatives.

**Table 2:** Heatmap of the stakeholder groups’ barrier preferences.

Grey – Answered by less than 50% of participants from the stakeholder group; White – Ranked 10th or lower within the stakeholder group; Yellow – Ranked between 7th and 9th within the stakeholder group; Orange – Ranked between 4th and 6th within the stakeholder group; Red – Ranked among the top 3 within the stakeholder group. DR – drug repurposing; HTA – health technology assessment; MAH – market authorisation holder, SME – small medium-sized enterprise.

## 4 Discussion

### 4.1 Priority barriers and stakeholders’ perspectives

The participants’ responses highlighted the fact that the consortium’s broad definition of DR encompasses a wide range of scenarios. Besides new indication for off-patent drugs, on-patent label extensions or repurposing shelved products are considered as DR. Some barriers are specific to particular types of DR, and even the generalizable barriers have different constraints on different RMs (e.g. barriers related to generic pricing mechanism do not apply to original products). The shortlisted key barriers identified through the survey were commonly emphasized during the prior validation meetings as well as in the textual comments of the survey and are also frequently mentioned in the literature [9], [10], [18], [19], [20], [21], [22], [23], [24], [25], [26], [27], [28].

The survey results indicated that some barriers had a higher relative impact on DR, while most of them were rated low for actionability (see Fig. 2 & Table 1). Some barriers are perceived as ‘low-hanging fruits’ (i.e. easier-to-tackle barriers), such as the limited know-how for SMEs (#21); multi-stakeholder collaboration (#20); or awareness of evidence requirements (#10). However, some highly impactful barriers were considered not easy to overcome, like the ones related to current pricing mechanisms (#1, #7), or legal aspects of DR (#2, #16, #18) [18], [19]. Survey results (see Table 2) also confirmed earlier observations from the validation meetings that the different stakeholder groups had different perceptions of the barriers.

#### 4.1.1 Barriers related to pricing

Three stakeholder groups out of the five – HTA, healthcare payer and regulator experts, pharmaceutical industry and researchers – marked the current (generic) pricing mechanism (#1) as one of the main hurdles of DR (the remainder two stakeholder groups had less than 50% response rate for the barrier). Multiple stakeholders mentioned that a fair price (i.e. differential pricing per indication or price premium compared to generics) for RMs is necessary to ensure financial return for innovators and secure their business case. Indication-based pricing (both for off-patent and on-patent RMs) may be a potential way forward; however, this approach – in case of off-patent pharmaceuticals – also implies indication-based prescription to avoid free ridership of manufacturers other than the investor of the off-patent RM (#5) [20]. Nevertheless, indication-based pricing was considered to be problematic (#7) [19]. For example, it may disrupt external reference pricing and be undermined by parallel trade – where lower-priced packs for one indication are re-exported and used for higher-priced indications elsewhere, weakening price differentials.

#### 4.1.2 Barriers related to legal issues

Another highly prioritized barrier and frequently mentioned problem was the legal protection of the RMs (#2, #19) [10]. This was ranked the second most important barrier by the pharmaceutical industry representatives. Participants stated that besides the limitation of market protection, the other important barrier is the cost of patenting as well as the difficulty of the enforcement of patent protection (#16) [10].

#### 4.1.3 Barriers related to the regulatory pathway

If the compound is still under patent protection (e.g. in case of shelved medicines), the need to engage with the MAH (#9) was identified as one of the main barriers by both researchers and funders [25]. The recent report of the EMA repurposing pilot also describes unsuccessful attempts of cooperation between the non-profit or academic developers and the MAH [29]. This issue, together with the challenges of generating the required evidence for regulatory approval- (#12, #15) and HTA application (#8, #13) was extensively mentioned by participants and also emphasized in the literature [24], [27]. However, stakeholders expressed differing views on the latter, with SMEs and researchers expressing more difficulties compared to manufacturers (often with dedicated in-house professional expertise) regarding the regulatory pathway and requirements (also represented in their top of stakeholder priority list); while “burdensome HTA requirements not designed for RMs” (#13) was the most important barrier for manufacturers. Reducing evidence requirements (i.e. lowering regulatory standards) was also perceived differently by government bodies (i.e. regulators) and innovators (i.e. researchers), partly due to potential safety concerns. The lack of clarity and awareness regarding evidence requirements (#10) [26] was also ranked high by the Funders. It was also noted that, while specific regulatory incentives already exist for certain categories of drug development, such as for orphan drugs, these are not universally applicable to all DR projects.

#### 4.1.4 Barriers related to funding

Beyond the necessary involvement of the MAH and lack of clarity in evidence requirements, funders identified fragmented and insufficient funding (#6) [22], [23] as their most important barriers. The recent initiative by the EMA aiming to improve clarity on evidence requirements by giving early scientific advice also acknowledged the lack of funding as one of the main barriers [29]. Survey respondents raised the question of whether limited funding is a consequence of the high failure rates or whether many projects fail because of the lack of funding. In the survey some funders noted that the available financial support often runs out after proof-of-concept studies, leaving insufficient resources to progress to later phase I-II clinical trials. The question was also raised in the comments whether, in the absence of for-profit investors, the government could provide funding for these projects, since society as a whole could benefit from DR initiatives. Unfortunately, the policy survey also showed that public–private partnerships in funding DR research are complex and not always possible (#20) [28].

#### 4.1.5 Barriers related to off-label use

The HTA, healthcare payer, and regulator stakeholder groups identified that, beyond the application of generic pricing mechanisms, the lack of incentives to turn off-label to on-label use (#3) was one of the most critical barriers. While off-label use can provide a limited number of patients with access to otherwise unavailable treatments, it also raises safety concerns due to lack of manufacturer’s support services, beyond access issues (as off-label use, even if permitted, is often not reimbursed) [30]. The working group within the REMEDi4ALL Consortium considers achieving on-label market authorization as an optimal endpoint of a successful DR project, to maximize societal return. However, in their comments, public funders noted that some DR projects they support often reach only off-label use stage rather than the more desirable on-label use (achieved through formal market authorization) as an outcome. This issue is closely linked to another key barrier: the cost of DR development is perceived to be disproportionately high compared to the risks and potential revenues (#18) [9], especially by non-pharma innovators. Funders confirmed that, in some cases, they deliberately opt for off-label use because pursuing on-label authorization and enforcement of market protection due to cross-label prescribing and dispensing would be too costly (#4, #11) [27].

#### 4.1.6 Perception of patient representatives

Patient representatives assessed the most important barriers very differently from those of other stakeholders. They considered that the general perception of off-patent DR compared to de novo drug discovery (#17) could be an important barrier. It was emphasized in their comments that there is strong expectation from both governments and society for new (original) drug discoveries, which may undermine other research areas. It was also stated that the general perception of off-patent DR needs to change among all stakeholder groups, including patients. Patient representatives identified the lack of know-how for DR for non-profit organizations as their top barrier (#21) [26]. Indeed, the REMEDi4ALL consortium aims to solve this issue by providing accessible educational materials as well as improving the literacy on DR at a broader level for patient representatives, researchers and the public, also to tackle perception-related barriers [31]. Finally, patient representatives also considered the lack of willingness from pharmaceutical companies to file label extensions for on-patent medicines due to return-on-investment and strategic business decisions (#22) among their top barriers [21].

### 4.2 Impact and next steps of the research

As far as we are aware, previous reviews about DR barriers have not attempted to prioritize them. This research, built on the DR barrier list of the prior SLR, pursued to identify the most important barriers. The barrier list prioritized by the recent policy survey will provide additional input for developing policy interventions to improve the DR ecosystem.

Subsequently, facilitators of DR also identified and extracted during the prior SLR will be collected into a Catalogue of Actions. This research step will be supplemented with collecting prior good practices of R&D incentives from other areas beyond DR, and experience from successful DR reference cases [32]. Then the identified facilitators will be validated and matched with the shortlisted barriers to ensure that the recommendations sufficiently cover the most important barriers. Finally, the interventions will also be assessed from a transferability perspective, to ensure their applicability in lower income EU countries (see Fig. 1).

The study also demonstrated that there is a considerable difference in the perception of DR barriers across different stakeholder groups while highlighting patients’ perspectives, which remain a central concern and mission for the consortium. Therefore, future research by the REMEDi4ALL Consortium focusing on both policy recommendations improving the DR ecosystem and on their transferability assessment also needs to be based on multi-stakeholder collaboration aiming for consensus.

### 4.3 Limitations

Besides the fact that individual perspectives may differ for the listed barriers, different interpretations of them could have influenced the scoring. The conversion of the categorical scales into 0–4 scores assumes that the difference among categories were equal, which was necessary from the feasibility point of view, but should be noted as an important limitation. While the calculated composite scores for the barriers facilitated their shortlisting, and shortlisting rules were explicit and justified, they were also set up somewhat arbitrarily. Thus, we emphasize the shortlist of barriers as the main output of the policy survey, rather than focusing on the individual impact and actionability scores of each barrier. While all stakeholder groups were represented in the policy survey, the sizes of stakeholder groups varied, and there was a large difference between the largest and smallest groups (6 and 17 respondents); nevertheless, statistical significance and representativity would not be a realistic objective of such a policy study in the field of DR. While the research aims to focus on the DR policy landscape in Europe, we also collected responses from participants outside the EU (i.e. UK, Others), assuming they have relevant knowledge of the European context. Indeed, we believe that the relatively high number of responders is a strength of this policy research. Finally, the design effect (i.e. response rate tends to decrease along with proceeding in the survey themes) was not mitigated by randomizing the order of items (themes), as the barriers were presented in an order reflecting the R&D process of medicines.

## 5 Conclusion

The policy study reinforced the relevance of the policy barriers to DR identified in the SLR. It highlighted the fact that the current DR landscape is characterized by multiple challenges, each varying in terms of how impactful and actionable they are as perceived by different stakeholder groups. Barriers considered to be the most important by responders relate to existing pricing structures, the challenges of securing patents and legal protection for off-patent innovations, and the absence of a viable business case for such repurposing efforts. The findings also confirmed that there are multiple types of DR, each facing distinct policy hurdles with varying levels of perceived impact.

Finally, the quantitative nature of our survey and analysis revealed that different stakeholder groups can perceive the relative importance of even the top barriers quite differently. This runs counter to the common assumption that there is a simple and linear rank order of barriers to DR that could be addressed one after the other (e.g., starting with the barrier of highest importance). This complex and close interplay adds critical multidimensionality to solution generation, as reducing a barrier for one stakeholder group may inadvertently raise another barrier for another stakeholder group. These findings highlight the overall heterogeneity and complexity of DR and that further investigations to develop strategies to address these barriers must always engage and involve multiple, preferably all, impacted stakeholder groups. Education and communication among different stakeholders will thus be essential to elucidate differences in how they understand each barrier and ultimately in the development of potential policy solutions.

## Funding Statement

This work was supported by the REMEDi4ALL project, which has received funding from the European Union’s Horizon Europe research and innovation programme under grant agreement No 101057442. Views and opinions expressed are those of the author(s) only and do not necessarily reflect those of the European Union, who cannot be held responsible for them.

## CRediT authorship contribution statement

**Kristóf Gyöngyösi:** Writing – review & editing, Writing – original draft, Project administration, Methodology, Investigation, Formal analysis, Data curation, Conceptualization. **Zsuzsanna Ida Petykó:** Writing – review & editing, Validation, Methodology, Funding acquisition, Conceptualization. **Dalma Hosszú:** Writing – review & editing. **Pan Pantziarka:** Writing – review & editing. **Helene G. van der Meer:** . **Donald C. Lo:** Writing – review & editing. **Marcell Csanádi:** Writing – review & editing. **George Dennis Obeng:** Writing – review & editing. **Zoltán Kaló:** Writing – review & editing, Supervision, Methodology, Funding acquisition. **András Inotai:** Writing – review & editing, Validation, Supervision, Methodology, Conceptualization.

## Declaration of competing interest

The authors declare the following financial interests/personal relationships which may be considered as potential competing interests: Kristóf Gyöngyösi, Dalma Hosszú, Marcell Csanádi, Zoltán Kaló and András Inotai are employees of Syreon Research Institute (SRI). Zsuzsanna Ida Petykó was an employee of SRI at the time the research was conducted. SRI received funding for studies on DR/value-added medicines from Egis Pharmaceuticals PLC and Medicines for Europe. All other co-authors declared no conflict of interest.

## References

[1] Ashburn T.T., Thor K.B. (2004). Drug repositioning: identifying and developing new uses for existing drugs. Nat Rev Drug Discov.

[2] Berdigaliyev N., Aljofan M. (2020). An overview of drug discovery and development. Future Med Chem.

[3] Wouters O.J., McKee M., Luyten J. (2020). Estimated research and development investment needed to bring a new medicine to market, 2009-2018. J Am Med Assoc.

[4] Lombardo S.D., Basile M.S., Ciurleo R., Bramanti A., Arcidiacono A., Mangano K. (2021). A network medicine approach for drug repurposing in duchenne muscular dystrophy. Genes (Basel).

[5] Morselli Gysi D., do Valle Í., Zitnik M., Ameli A., Gan X., Varol O., Ghiassian S.D., Patten J.J., Davey R.A., Loscalzo J., Barabási A.L. (2021). Network medicine framework for identifying drug-repurposing opportunities for COVID-19. PNAS.

[6] *REPurpOsing For EUrope and the world Data Hub*. Available from: https://repo4.eu/the-platform/. [accessed 9 October 2025].

[7] *Reactome*. Available from: https://reactome.org/. [accessed 9 October 2025].

[8] *DisGeNET*. Available from: https://disgenet.com/. [accessed 9 October 2025].

[9] Roessler H.I., Knoers N., van Haelst M.M., van Haaften G. (2021). Drug repurposing for rare diseases. Trends Pharmacol Sci.

[10] Krishnamurthy N., Grimshaw A.A., Axson S.A., Choe S.H., Miller J.E. (2022). Drug repurposing: a systematic review on root causes, barriers and facilitators. BMC Health Serv Res.

[11] *REPurpOsing For EUrope and the world*. Available from: https://repo4.eu/. [accessed 9 October 2025].

[12] *REMEDi4ALL, Repurposing of Medicines 4 All*. Available from: https://remedi4all.eu/. [accessed 9 October 2025].

[13] *DrugTrain, European Training Network on Drug Repurposing.* Available from: https://drugtrain.eu/. [accessed 11 February 2026].

[14] *DREAMS Horizon Project, Drug Repurposing Ecosystem through AI and Model Sharing*. Available from: https://dreamshorizon.eu/. [accessed 9 October 2025].

[15] *SIMPATHIC, Simulation-Guided Pathways for Efficient Drug* Repurposing. Available from: https://simpathic.eu/. [accessed 9 October 2025].

[16] Bouygues C., Tavridou A., Herold R., Nuevo-Ordoñez Y. (2026). Stimulating medicines repurposing in the EU: a pilot project. Nat Rev Drug Discov.

[17] Petykó Z.I., Hosszú D., Csanádi M., Gyöngyösi K., Obeng G.D., Ameyaw D. (2025). The eyes of the beholder: perceived barriers to successful drug repurposing. Brit J Phramacol.

[18] Cummings J.L., Zhong K. (2014). Repackaging FDA-approved drugs for degenerative diseases: promises and challenges. Exp Rev Clin Pharmacol.

[19] Toumi M., Rémuzat C. (2017). Value added medicines: what value repurposed medicines might bring to society?. J Mark Access Health Policy.

[20] Petykó Z.I., Inotai A., Holtorf A.P., Brixner D., Kaló Z. (2020). Barriers and facilitators of exploiting the potential of value-added medicines. Expert Rev Pharmacoecon Outcomes Res.

[21] Antolin A.A., Workman P., Mestres J., Al-Lazikani B. (2016). Polypharmacology in precision oncology: current applications and future prospects. Curr Pharm Des.

[22] Del Álamo M., Bührer C., Fisher D., Griese M., Lingor P., Palladini G. (2022). Identifying obstacles hindering the conduct of academic-sponsored trials for drug repurposing on rare-diseases: an analysis of six use cases. Trials.

[23] Giovannoni G., Baker D., Schmierer K. (2015). The problem with repurposing: is there really an alternative to big pharma for developing new drugs for multiple sclerosis?. Mult Scler Relat Disord.

[24] Shineman D.W., Alam J., Anderson M., Black S.E., Carman A.J., Cummings J.L. (2014). Overcoming obstacles to repurposing for neurodegenerative disease. Ann Clin Transl Neurol.

[25] Siegelin M.D., Schneider E., Westhoff M.A., Wirtz C.R., Karpel-Massler G. (2021). Current state and future perspective of drug repurposing in malignant glioma. Semin Cancer Biol.

[26] Verbaanderd C., Rooman I., Meheus L., Huys I. (2019). On-label or off-label? overcoming regulatory and financial barriers to bring repurposed medicines to cancer patients. Front Pharmacol.

[27] Zhang H., Zaric G.S. (2015). Using price-volume agreements to manage pharmaceutical leakage and off-label promotion. Eur J Health Econ.

[28] Verbaanderd C., Rooman I., Huys I. (2021). Exploring new uses for existing drugs: innovative mechanisms to fund independent clinical research. Trials.

[29] *EU Repurposing pilot* Available from: https://www.ema.europa.eu/en/documents/report/eu-repurposing-pilot_en.pdf. [accessed 11 February 2026].

[30] Lenk C. (2012). Off-label drug use in paediatrics: a world-wide problem. Curr Drug Targets.

[31] *REMEDi4ALL Repurposing Academy, Resource hub for drug repurposing training*. Available from: https://remedi4all.org/resources/remedi4all-repurposing-academy/. [accessed 9 October 2025].

[32] Inotai A., Hosszú D., Gyöngyösi K., Pozsar Z.R., Winckers M., Lo D. (2025). P51 catalogue of actions to address policy barriers of drug repurposing. Value Health.

