# Supplementary material for: Policy barriers to drug repurposing in Europe: different stakeholder perspectives identified during survey-based shortlisting of key challenges
Source: Health Policy Open. 2026 Mar 25;10:100168. doi: 10.1016/j.hpopen.2026.100168 (PMC13068566; doi:10.1016/j.hpopen.2026.100168)
Supplement: Supplementary Data 1 [file mmc1.docx]

Supplementary Table 1 Outline of the main survey section

| Main theme | Subtheme | Barrier | Impact: | | | | | Actionability: | | | | |
| --- | --- | --- | --- | --- | --- | --- | --- | --- | --- | --- | --- | --- |
|  |  |  | negligible | minor | moderate | major | extreme | very poor | poor | average | easy | very easy |
| Is the topic of Perception of repurposing of off-patent medicines within your expertise? | | | Yes No | | | | | | | | | |
| I. Perception of repurposing of off-patent medicines | Perception of off-patent DR | 1. DR research of off-patent medicines is perceived as less innovative, less robust or less attractive compared to de novo drug development. |  |  |  |  |  |  |  |  |  |  |
|  |  | 2. Repurposed off-patent medicines may be perceived to be inferior compared to de novo medicines. |  |  |  |  |  |  |  |  |  |  |
|  |  | 3. The cost of DR development is perceived to be disproportionally high compared to the risks and potential revenues. |  |  |  |  |  |  |  |  |  |  |
| (optional)  Beyond those listed above, would you like to propose any missing barrier(s) within the topic, or suggest any changes regarding the phrasing of the current barriers? If yes, please specify below: | | |  | | | | | | | | | |
| Is the topic of Business case for repurposing on-patent compounds within your expertise? | | | Yes No | | | | | | | | | |
| II. Business case for repurposing on-patent compounds | Incentives for originators | 4. Originator companies often lack incentives to repurpose on-patent compounds due to low expected return on investment and strategic business decisions regarding their disease portfolio. |  |  |  |  |  |  |  |  |  |  |
|  | Data exclusivity related to patented compounds | 5. Exclusive rights of originator companies to previously generated data blocks repurposing patented compounds for other companies. |  |  |  |  |  |  |  |  |  |  |
| (optional)  Beyond those listed above, would you like to propose any missing barrier(s) within the topic, or suggest any changes regarding the phrasing of the current barriers? If yes, please specify below: | | |  | | | | | | | | | |
| Is the topic of Business case for repurposing off-patent medicines within your expertise? | | | Yes  No | | | | | | | | | |
| III. Business case for repurposing off-patent medicines | Free ridership by cross-label use | 6. Competitors can benefit from the DR investment in the case of off-patent medicines by cross-label prescribing and dispensing. |  |  |  |  |  |  |  |  |  |  |
|  | Business potential related to repurposing of off-patent medicines | 7. Insufficient return on investment is anticipated for repurposing off-patent medicines. |  |  |  |  |  |  |  |  |  |  |
| (optional)  Beyond those listed above, would you like to propose any missing barrier(s) within the topic, or suggest any changes regarding the phrasing of the current barriers? If yes, please specify below: | | |  | | | | | | | | | |
| Is the topic of Non-industry funded DR within your expertise? | | | Yes  No | | | | | | | | | |
| IV. Non-industry funded DR | Interest in non-profit funding of DR | 8. There may be limited interest in non-profit funding of DR due to lack of incentives and uncertain path to patients. |  |  |  |  |  |  |  |  |  |  |
|  | Availability of funding for DR | 9. Limited, incomplete and fragmented funding is available for non-profit DR at different stages. |  |  |  |  |  |  |  |  |  |  |
|  | Funding DR via specific models | 10. Crowdfunding of DR is challenging, not suitable for large trials and relies on individuals’ sentiments. |  |  |  |  |  |  |  |  |  |  |
|  |  | 11. Public-private partnerships in funding DR are complex and not always possible. |  |  |  |  |  |  |  |  |  |  |
|  |  | 12. Applying pay-for-success models (e.g. social impact bonds) in funding DR and measuring its impact is difficult |  |  |  |  |  |  |  |  |  |  |
| (optional)  Beyond those listed above, would you like to propose any missing barrier(s) within the topic, or suggest any changes regarding the phrasing of the current barriers? If yes, please specify below: | | |  | | | | | | | | | |
| Is the topic of Ecosystem for non-profit or SME-driven drug repurposing within your expertise? | | | Yes No | | | | | | | | | |
| V. Ecosystem for non-profit or SME-driven drug repurposing | Infrastructure and data | 13. Lack of certified and established manufacturing infrastructure hinders non-profit or SME-driven DR projects. |  |  |  |  |  |  |  |  |  |  |
|  |  | 14. Lack of findable, accessible, interoperable, and reusable (FAIR) data (especially proprietary data) for DR. |  |  |  |  |  |  |  |  |  |  |
|  | Capabilities and resources | 15. The know-how needed for DR may not be available for non-profit entities or SMEs. |  |  |  |  |  |  |  |  |  |  |
|  |  | 16. Lack of multidisciplinary and multi-stakeholder collaboration in DR research. |  |  |  |  |  |  |  |  |  |  |
|  |  | 17. Limited capabilities and resources of non-profit entities and SMEs for branding and marketing of repurposed medicines. |  |  |  |  |  |  |  |  |  |  |
| (optional)  Beyond those listed above, would you like to propose any missing barrier(s) within the topic, or suggest any changes regarding the phrasing of the current barriers? If yes, please specify below: | | |  | | | | | | | | | |
| Is the topic of Market Authorisation within your expertise? | | | Yes  No | | | | | | | | | |
| VI. Market Authorisation | Evidence generation for authorizing repurposed medicines | 18. There is a lack of clarity/limited awareness on evidence requirements for some off-patent drug repurposing cases. |  |  |  |  |  |  |  |  |  |  |
|  |  | 19. Evidence generation is burdensome for the market authorisation of off-patent medicines. |  |  |  |  |  |  |  |  |  |  |
|  |  | 20. Incentives for market authorization of repurposed medicines in paediatric indications are not proportionate to the required efforts for evidence generation. |  |  |  |  |  |  |  |  |  |  |
|  | Market authorisation pathway for DR | 21. It is hard to navigate the multiple market authorisation pathways that are applicable for DR, which makes the process burdensome. |  |  |  |  |  |  |  |  |  |  |
|  |  | 22. For label extension, there is a need for the marketing authorisation holder’s involvement for non-MAH developers. |  |  |  |  |  |  |  |  |  |  |
| (optional)  Beyond those listed above, would you like to propose any missing barrier(s) within the topic, or suggest any changes regarding the phrasing of the current barriers? If yes, please specify below: | | |  | | | | | | | | | |
| Is the topic of Exclusivity rights within your expertise? | | | Yes  No | | | | | | | | | |
| VII. Exclusivity rights | Off-label use | 23. Limited incentives to turn off-label use to on-label to ensure access to a wider patient population. |  |  |  |  |  |  |  |  |  |  |
|  | Regulatory protection | 24. Limited market protection and data exclusivity options for repurposed medicines. |  |  |  |  |  |  |  |  |  |  |
|  |  | 25. Enforcement of market protection for repurposed medicines is difficult because of cross-label prescribing and dispensing. |  |  |  |  |  |  |  |  |  |  |
|  | Patent protection | 26. High cost of successful drug patenting in DR because of the increased time and resources needed to prove novelty. |  |  |  |  |  |  |  |  |  |  |
|  |  | 27. Limited options for patent protection of repurposed medicines. |  |  |  |  |  |  |  |  |  |  |
|  |  | 28. Enforcement of patent protection for repurposed medicines is difficult and costly. |  |  |  |  |  |  |  |  |  |  |
| (optional)  Beyond those listed above, would you like to propose any missing barrier(s) within the topic, or suggest any changes regarding the phrasing of the current barriers? If yes, please specify below: | | |  | | | | | | | | | |
| Is the topic of Health technology assessment (HTA) within your expertise? | | | Yes  No | | | | | | | | | |
| VIII. Health technology assessment (HTA) | Scientific evidence for HTA | 29. Evidence requirement for HTA is not designed for off-patent DR and is of high burden. |  |  |  |  |  |  |  |  |  |  |
|  | HTA process | 30. No tailored or predictable technology appraisal process exists for off-patent DR. |  |  |  |  |  |  |  |  |  |  |
| (optional)  Beyond those listed above, would you like to propose any missing barrier(s) within the topic, or suggest any changes regarding the phrasing of the current barriers? If yes, please specify below: | | |  | | | | | | | | | |
| Is the topic of Pricing within your expertise? | | | Yes  No | | | | | | | | | |
| IX. Pricing | Price potential of off-patent DR | 31. Generic pricing mechanisms are often applied to off-patent repurposed medicines. |  |  |  |  |  |  |  |  |  |  |
|  |  | 32. Disproportionally high prices of single-sourced repurposed medicines could hinder wide patient access. |  |  |  |  |  |  |  |  |  |  |
|  | Differential pricing | 33. Indication-based differential pricing for repurposed medicines is problematic. |  |  |  |  |  |  |  |  |  |  |
| (optional)  Beyond those listed above, would you like to propose any missing barrier(s) within the topic, or suggest any changes regarding the phrasing of the current barriers? If yes, please specify below: | | |  | | | | | | | | | |

*DR – drug repurposing; HTA – Health Technology Assessment; SME - Small medium-sized enterprises*

Supplementary Table 2 Characteristics of the participants

| Total participants | 60 | 100% |
| --- | --- | --- |
| Participants by stakeholder group | |  |
| HTA, healthcare payer, regulator | 11 | 18.3% |
| Patient representatives | 6 | 10.0% |
| Funders (Philanthropic or public funder of DR) | 12 | 20.0% |
| Pharmaceutical industry (pharmaceutical companies, biotech and SME, industry association, consultant, venture capitalist) | 17 | 28.3% |
| Researchers (researchers, academia and clinicians) | 14 | 23.3% |
| DR Expertise/perspective of participants by geographical distribution | |  |
| Old EU Member States (EU15 countries excl. UK) | 31 | 51.7% |
| New EU Member States (EU13 countries) | 12 | 20.0% |
| UK | 10 | 16.7% |
| Others (e.g. USA, Switzerland, Ukraine) | 7 | 11.7% |

*HTA - health technology assessment; SME - small-medium sized enterprises; EU - European Union; USA - United States of America; UK - United Kingdom*


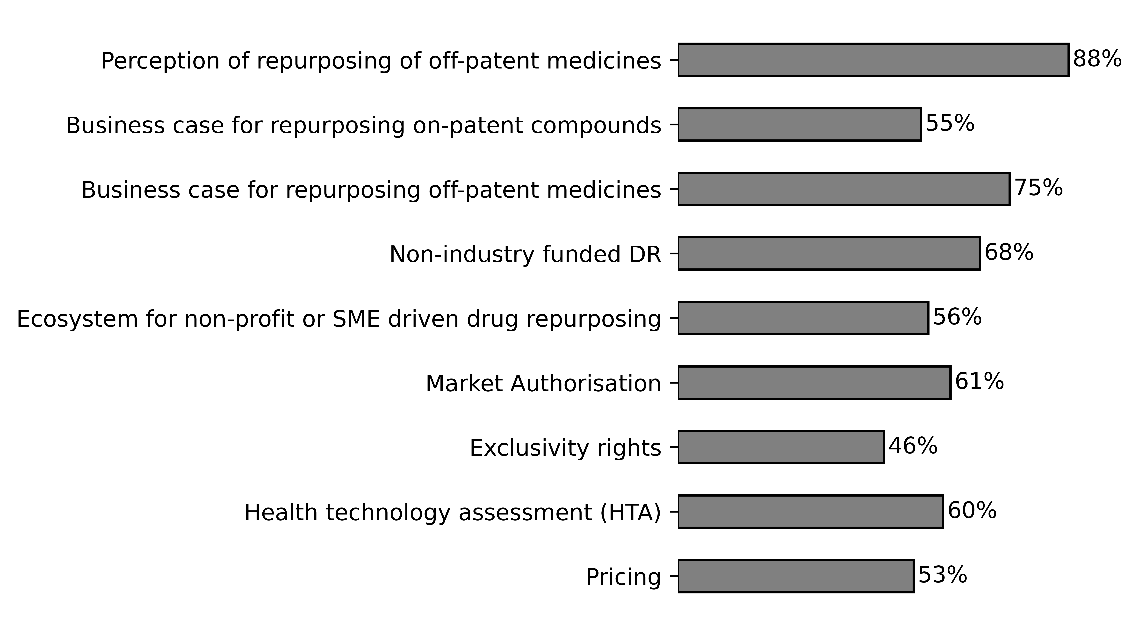


Supplementary Figure 1 Response rates for each theme, in order of their appearance in the survey (% of all respondents).

*DR - drug repurposing; HTA - health technology assessment; SME - small medium-sized enterprises*
